# Rapid Detection of *Clostridium tetani* by Recombinase Polymerase Amplification Using an Exo Probe

**DOI:** 10.4014/jmb.2109.09022

**Published:** 2021-11-24

**Authors:** Mingjing Guo, Pan Feng, Liqun Zhang, Chunfeng Feng, Jie Fu, Xiaoyun Pu, Fei Liu

**Affiliations:** Department of clinical laboratory, Xinqiao Hospital and The Second Affiliated Hospital, Army Medical University, No. 183 Xinqiao Main St, Shapingba District, Chongqing 400037, P.R. China

**Keywords:** Recombinase polymerase amplification, TeNT gene, rapid detection

## Abstract

Tetanus is a potentially fatal public health illness resulted from the neurotoxins generated by *Clostridium tetani*. *C. tetani* is not easily culturable and culturing the relevant bacteria from infected wounds has rarely been useful in diagnosis; PCR-based assays can only be conducted at highly sophisticated laboratories. Therefore, a real-time recombinase polymerase amplification assay (Exo-RPA) was constructed to identify the fragments of the neurotoxin gene of *C. tetani*. Primers and the exo probe targeting the conserved region were designed, and the resulting amplicons could be detected in less than 20 min, with a detection limit of 20 copies/reaction. The RPA assay displayed good selectivity, and there were no cross-reactions with other infectious bacteria common in penetrating wounds. Tests of target-spiked serum and pus extract revealed that RPA is robust to interfering factors and has great potential for further development for biological sample analysis. This method has been confirmed to be reliable for discriminating between toxic and nontoxic *C. tetani* strains. The RPA assay dramatically improves the diagnostic efficacy with simplified device architecture and is a promising alternative to real-time PCR for tetanus detection.

## Introduction

In humans, tetanus is a neurological disease with considerable mortality that results from tetanus neurotoxin (TeNT) production by toxigenic strains of *Clostridium tetani* (*C. tetani*) proliferating in a contaminated open wound [1]. *C. tetani* is a gram-positive, obligately anaerobic and spore-forming bacterium. Based on its ability to produce the thermolabile toxin, it can be mainly categorized into toxigenic strains and nontoxigenic derivatives. Previous research indicates that the structural gene coding for the neurotoxin is on a plasmid [1, 2]. The neurotoxin can be endocytosed by both Rab5- and clathrin-dependent mechanisms and then transported to the central nervous system (CNS) by long-distance retrograde axonal transport, where it can inhibit the release of neurotransmitters in the spinal cord, leading to periodic hyper contraction of skeletal muscles (termed spastic paralysis) [3]. The spores of *C. tetani* are abundantly found in soil and other environments; in most cases, tetanus originates from damaged skin or narrow wounds, such as those resulting from dental infections, intravenous drug abuse, compound fractures, or nail punctures. These areas confer a more favorable anaerobic environment for the growth and reproduction of *C. tetani* [4, 5]. Tetanus can be prevented by active immunization of children and pregnant women. There is no effective treatment for this disease. Tetanus management involves aggressive airway management, treatments to inhibit the absorption of toxins, strict sanitary measures, treatments to alleviate muscle spasms, high-intensity therapy, and intensive supportive care [6, 7]. Tetanus generally occurs in low and middle-income countries but has a low incidence in developed countries [6, 8]. Maternal and neonatal tetanus (MNT) maintains a widespread public health issue, with mortality between 80% and 100% among neonates [9]. It is estimated that one million cases of tetanus happen worldwide annually and led to between 48199 and 80042 deaths in 2015. The World Health Organization (WHO) estimated that 30,848 newborns died of neonatal tetanus in 2017, and the imputed number of neonatal tetanus deaths decreased to 25000 in 2018 [10, 11]. Although mortality is steadily declining, we believe that the global incidence of the disease is underestimated because most cases arise in resource-limited settings where surveillance systems are limited, and accurate data cannot be obtained. Over the past few decades, intensive care measures have undergone significant changes in numerous countries, and it is possible that high tetanus incidence rates persist despite the remarkable declines in mortality that have occurred [12-14]. This is an additional factor that may result in an underestimation of disease.

The diagnosis of tetanus typically relies on the clinical history and major symptoms. In localized tetanus, differential diagnosis is necessary, and laboratory tests can exclude any other diseases with similarities to tetanus, such as strychnine poisoning, neuroleptic malignant syndrome, stiff-person syndrome, and dystonic reaction to drugs. Without medical attention, the mortality rate of neonatal tetanus approaches 100%, and it often surpasses 50% even with treatment [13, 15]. Early diagnosis of *C. tetani* can substantially prevent or interfere with the progression of the disease. Commonly, the gold standard for verification of bacteria is the bacterial plate culture, which needs at least 3-7 days to obtain a result [16]. *C. tetani* is an obligate anaerobic bacterium, the culture-based diagnostic method is not optimal, anaerobic cultivation is time-consuming, and this method requires relatively advanced laboratory conditions. Moreover, not all patients contaminated with *C. tetani* will develop tetanus; therefore, it is necessary to distinguish between toxic and nontoxic strains. In the past, guinea pigs or mice were commonly employed to assess the potent toxicity of tetanus toxoid, but this method had low ethical and economic acceptability [17, 18]. At present, many technologies have been constructed to estimate tetanus toxoid in vitro, such as fluorescent immunochromatography test strips [18] and BINACLE (binding and cleavage) assay [19]. The abovementioned methods present a few disadvantages, including relatively low sensitivity and specificity, and cannot provide accurate quantification. Various quantitative approaches have been established, like real-time polymerase chain reaction (PCR) [20] and loop-mediated isothermal amplification assays (LAMP) [21], to determine the fragments of the TeNT gene. PCR-based assays require automated thermocycling equipment and trained personnel, which limit their potential clinical applications in resource-poor regions. Therefore, it is extremely necessary to build an advanced method.

Various isothermal nucleic acid amplification assays have been designed as substitutes for PCR [22-24]. Because the amplification process relies on enzymatic processes at all stages, these methods can be carried out under mild conditions (*e.g.*, chemical heater, heating block), on the human wrist, dorsum manus [25, 26], and even within living cells [27], which is unattainable through PCR. Numerous detection systems integrate isothermal amplification with ultraportable platforms or microsystems to achieve field visualization capability and offer high sensitivity and specificity. Recombinase polymerase amplification (RPA) is one of these assays [28], which can achieve rapid accumulation of products using three principal enzymes: recombinase (T4 UvsX), single-stranded DNA-binding proteins (SSB), and DNA polymerase (Bsu). Less than 20 min is required to complete the whole amplification process at 37-42°C, eliminating the requirement for a thermocycler. Real-time monitoring of the amplification depends on the application of *E. coli* exonuclease III, which can identify and shear the abasic site (*e.g.*, tetrahydrofuran, THF), with a detection limit as low as 1-10 copies/μl. RPA appears to be a promising method for detecting multiple different biological targets [28, 29]. RPA has been successfully applied to fields as diverse as pathogen identification [30-33], environmental monitoring [32], food testing [34, 35], and disease diagnostics [36]. RPAs combined with wearable flexible microfluidic devices [25, 26], solar-powered suitcase labs [34], i-chips and f-boxes [32] have been constructed, and these portable and user-friendly devices may provide a flexible and direct platform for future point-of-care tests.

In the present study, we demonstrated an Exo-RPA assay for amplifying specific TeNT gene fragments to aid in the diagnosis of toxic *C. tetani* infection. This method dramatically reduced the detection time, simplified the operation steps, and could be monitored by simple equipment. The whole amplification process could be completed in 20 min, and accurate results could be obtained from as little as 10 μl of reaction volume. The experimental results indicated that the proposed technology could serve as a potential alternative to PCR to fulfill the requirements of point-of-care testing.

## Materials and Methods

### Exo-RPA Assay

Exo-RPA trials were conducted by a TwistAmp Exo kit (TwistDx, United Kingdom). The reactions were conducted in 50 μl using the RPA lyophilized pellet, which offered all components required for DNA amplification. For the Exo-RPA assay, 29.5 μl of rehydration buffer was blended with 10.8 μl of ddH_2_O, 2.1 μl of forward/reverse primer (10 μM), 1 μl of exo probe (10 μM), and 2 μl of templates as the master mix. The total volume of 47.5 μl of the reaction solution was distributed into the tube of freeze-dried pellet. At the start of amplification, the catalytic agent was infused into the tube lids, and the tubes were closed carefully. Then, the isolated tandem reagent tubes were mixed to homogeneity, centrifuged, and placed in a tube scanner to ensure that the amplification reaction began simultaneously. All tandem tubes were kept at 39°C for 20 min, followed by fluorescence recording (Ex: 492 nm/Em: 518 nm) every 20 sec (ABI QuantStudio1, USA).

### Standard Samples and RPA Oligonucleotides

The TeNT gene was found to be encoded on a plasmid [2]. The targeting sequence of *C. tetani* was retrieved from GenBank, National Center for Biotechnology Information (NCBI). The specific nucleic acid sequence of the neurotoxin gene (No. AF154828.2) was subjected to conservation analysis by BioEdit v.5.0.9 (Tom Hall, North Carolina State University) and genetic homology analysis by the BLAST search tool. Eventually, the DNA standard was obtained by screening and cloning a 240 bp highly conserved fragment into the pUC57 plasmid (Sangon Biotech Co., China). We present the candidate DNA fragment in Table S1. The standard *C. tetani* strain (ATCC 19406) was purchased from the American Type Culture Collection Center (USA) and cultured in the Columbia blood agar (Pangtong, China). The standard strain of *C. tetani* (NCTC 5405) was purchased from the National Collection of Type Cultures (England) and inoculated into anaerobic bottles (Thermo Scientific, Germany). Both kinds of bacteria were identified as *C. tetani* by mass spectrometry (Bruker Microflex, Germany). Genomic DNA was extracted from each strain using the TaKaRa MiniBEST Bacteria Genomic DNA Extraction Kit Ver.3.0 (Takara, China).

Primer Premier Design 5.0 was used for specific RPA and PCR primer and probe design following the guidelines recommended in the TwistDx manufacturer kit instructions and universal rules. The probe was designed to accommodate all primer sets. Six primers and one exo probe were produced by Sangon Biotech and purified by PAGE and HPLC, respectively. The probe and the forward primer were arranged in the same direction to avoid possible primer-primer dimers and primer-probe dimers. The sequences of all the RPA oligonucleotides are tabulated in Table 1. In the selection process of the optimal primer pair, 9 combinations consisting of 3 forward and reverse primers were selected, and 10^5^ copies of recombinant pUC57 plasmid served as the template. After determining the best primer pair, the probe concentration (corresponding to volumes) was subsequently optimized by testing the following concentrations: 80 nM, 120 nM, 160 nM, 200 nM, 240 nM, and 280 nM (note: primer concentration was under the manufacturer’s recommendation of 420 nM).

### Sensitivity and Specificity

Recombinant plasmids were diluted by tenfold serial dilutions (10^0^-10^8^ DNA copies/μl) for sensitivity analysis. All serial dilutions were tested in three independent runs. Basic RPA amplicons were purified by a QIAquick PCR Purification Kit (Qiagen, Germany) and determined via 1.5% agarose gel electrophoresis. For visual fluorescence detection, 1 μl of Nuclear Staining Dyes (Solarbio, China) and 4 μl of amplification products were mixed, and the result was detected by Gel Doc XR+ (BIO-RAD). To evaluate the specificity of this strategy, eight strains of interfering bacteria were identified, including *Staphylococcus aureus*, *Staphylococcus epidermidis*, *Escherichia coli*, *Acinetobacter baumannii*, *Pseudomonas aeruginosa*, *Enterococcus faecium*, *Klebsiella pneumoniae*, and *Stenotrophomonas maltophilia*, and extracted genomic DNA was analyzed using a Nanodrop 2000 spectrophotometer (Thermo Scientific). The copy numbers of the bacterial genomes ranged from 2.6 × 10^6^ to 4.1×10^8^ copies/μl. These bacterial samples were collected from bacteriological cultures and kindly provided by the Xinqiao Hospital, Chongqing, China. All bacterial strains were cultured in the appropriate medium at 37°C for 24 h, and all were confirmed by matrix-assisted laser desorption ionization-time of flight mass spectrometry (Bruker Microflex, Germany). To preclude the presence of substances that inhibited amplification during bacterial DNA extraction, a positive control contained in the exo kit was injected into the reaction as an internal control. In the presence of extracted genomic DNA, a negative result for the internal control amplification suggested that the sample contained inhibitors of the RPA assay, and re-extraction was necessary; the opposite result indicated that the extracted nucleic acid was suitable for use.

### Real-Time PCRs

To evaluate the performance of our method, we compared it to the “gold standard” PCR method. Two PCR amplification primer sets were designed, and combination 2 (F2, R2, P2) was selected to validate the performance of the PCR assay. Tenfold gradient dilutions of the TeNT gene plasmids (10-10^8^ copies/μl) were used to verify the LOD of the PCR method. All the PCR studies were monitored by an Applied Biosystems QuantStudio Real-Time PCR System (USA). The reagents used were Premix Ex TaqTM (Takara). The final reaction volume of 20 μl comprised 10 μl of master mix, 0.4 μl of forward/reverse primer, 0.8 μl of probe, 0.2 μl of ROX Reference Dye II, 2 μl of templates, and 6.2 μl of nuclease-free water. The reaction conditions were 95°C for 30 s, followed by 40 cycles of 95°C for 5 s, 60°C for 34 s. The PCR primers and probes employed in the project are enumerated in Table S1.

### Repeatability and Influence of Different Volumes on the Performance of the Assay

Repeatability, as an essential indicator of the assay, was estimated by running parallel tests of the RPA with template copy numbers ranging from 10 to 10^5^ copies/μl. To investigate whether differences in volume would impact our results, we performed the assays in 10 μl, 15 μl, and 50 μl.

### Application of the Exo-RPA Assay in Real Sample

A critical issue to consider during practical application is the interference of protein interactions. A range of specimens with different concentrations of target (10^1^ to 10^3^ DNA molecules/μl) were added to healthy human serum to explore the influence of serum (containing a variety of proteins) on the experimental results. Infectious wounds might contain many pus cells and other bacteria; therefore, abundant background DNA existed after extracting the pus samples. To show the robustness of our RPA tests in the existence of background DNA, different concentrations of the TeNT gene were spiked into the pus-extracted samples, and the mean threshold time values were compared to those of the same batch of normal samples. Human serum and pus samples were obtained from Xinqiao Hospital. An optimized reaction system was developed to detect the toxic strain (NCTC 5405) and nontoxic strain (ATCC 19406).

### Data Analysis and Statistics

A baseline detection threshold was established to calculate the threshold time (TT), and the fluorescence threshold for a positive reaction was designated as 3 standard deviations plus the averaged fluorescence intensity values observed in ten no targets control (NTC) reactions. The TT for each standard concentration was designed as the time at which the fluorescence passed the calculated threshold value. The standard curve was constructed using the log values of the templates and the mean value of the TT corresponding to each concentration. Each abovementioned assay was performed 3 times, and error bars denote the standard deviation (SD).

## Results

### Screening of RPA Primer and Corresponding Optimal Probe Concentration

**Fig. 1:**
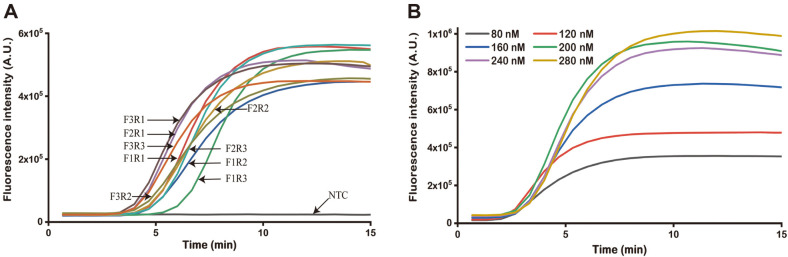
Screening primer combinations and probe concentration. (**A**) The primer pairs F1R1-F3R3 were used for tetanus toxin gene fragment amplification assays. (**B**) Real-time fluorescence curves of different concentrations of probes for amplification to detect targets.

Previously published studies showed that the neurotoxin gene contained on a single large plasmid, and we constructed a plasmid containing the conserved part of the TeNT gene as a standard DNA sample. For the RPA assays, the primers and probe were synthesized for amplification of the target fragments to avoid nonspecific effects. A total of nine primer-probe pairs were identified and screened for reactivity with standard DNA. All tested combinations were effectively amplified, and the amplification curves of the target were observed to have sigmoid shapes. However, only three of the pairs could attain the threshold over a short period, and primer set 7 resulted in an earlier amplification starting point than the other combinations (Fig. 1A). Consequently, primer set 7 (TeNT gene-F3-RPA and TeNT gene-R1-RPA) was adopted for further experiment confirmation. The effects of various concentrations of probe on the RPA signaling response were explored from 80-280 nM at a fixed primer concentration of 420 nM. As shown in Fig. 1B, the fluorescence intensity and the efficiency of the reaction increased progressively with increasing probe concentration up to 200 nM. Hence, a concentration of 200 nM was selected as the ideal concentration for all successive assays.

### Analytical Sensitivity of Exo-RPA for Nucleic Acids

**Fig. 2:**
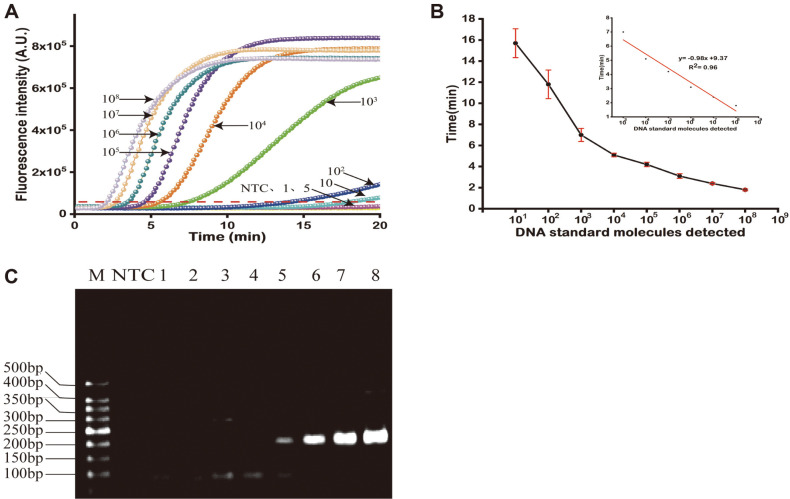
Experimental sensitivity of the Exo-RPA. (**A**) The fluorescence intensity correlates with the different target concentration. Different copy numbers of the TeNT gene were amplified by Exo-RPA. The threshold is represented by the red dotted line at 48788. (**B**) The correlation between threshold time and target TeNT gene content, 10^0^-10^8^ copies/μl. The error bars indicate the standard error of three independent experiments. The inset is the image of the calibration curve of threshold time vs. target concentration, 10^3^-10^8^ copies/μl. (**C**) Sensitivity experiment using basic recombinase polymerase amplification and a series of diluted templates. M: marker; NTC: no target control; 1-8: 10-10^8^ copies/μl.

Known quantities of target were employed to evaluate the lowest detection limit of the RPA assay. The sensitivity analysis is presented in Fig. 2A. The real-time fluorescence signal was detected at earlier time as the concentration of the target increased. When the target was added, the fluorescence intensity was exponentially increased compared with that of the negative control. RPA could detect a minimum concentration of TeNT gene of 20 copies/reaction indicating its high sensitivity for detecting targeted substrates. To analyze the quantitative capability of this method, a series of dynamic curves was plotted for each concentration of the target. The fluorescence threshold was expressed as the mean plus three times the standard deviation of the negative control, and the cutoff value was 48788. The relationship between the TT and the DNA concentrations under optimal conditions is illustrated Fig. 2B. From the results, we concluded that the threshold time decreased with increasing initial target concentration. Fig. 2B also shows the linear relationship between the threshold time and the logarithm of TeNT gene concentrations. The linear equation was y = -0.98x+9.37 (R^2^ = 0.96), where y was the threshold time and x was the logarithm of target DNA. The threshold time of the real-time RPA could be converted to the number of bacteria according to the standard curve. The concentration of DNA and its threshold time are listed in Table 2. As shown in Fig. 2C, a 196 bp band was obtained from the basic RPA reaction, while the negative control showed no amplification. From the electrophoresis, we could conclude that the sensitivity of electrophoresis was much lower than that of real-time fluorescence detection. The RPA assay data acquisition could be obtained by a highly specific exo probe and standard agarose gel electrophoresis. The experimental sensitivity of the PCR is displayed in Fig. S1 (plasmid).

### Specificity of Exo-RPA for Nucleic Acids

**Fig. 3:**
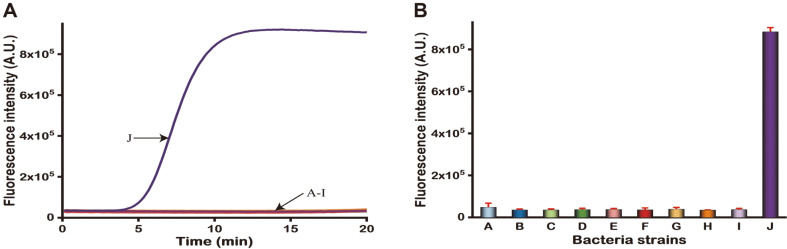
Analytical specificity of the Exo-RPA trial. (**A**) Exo-RPA assay shows no evidence of cross-reactivity, and only target genes are tested. (**B**) The histogram of fluorescence intensity of different bacterial genomes. Error bars indicate the standard error of three replicate experiments. A-J: *S. epidermidis*, *K. pneumoniae*, *P. aeruginosa*, *S. maltophilia*, *A. baumannii*, *E. coli*, *S. aureus*, *E. faecium*, NTC (using nuclease-free water as a negative control), and 10^5^ copies/μl recombinant plasmid DNA.

Specificity is an important element that must be assessed before using the RPA assay as a sensitive assay test. First, we verified the quality of the extracted DNA. The internal control was infused into the reaction tube, while the internal control was positive and the bacterial genome was negative, indicating that the DNA preparation was of sufficient quality. Then, we proceeded to the next step of verification. To investigate the specificity of the RPA assay, eight different bacterial genomes were tested as competitors. In these RPA assay tests, only the target DNA triggered significant exponential amplification, which could be observed by the fluorescence biosensing system, whereas all other genomic samples showed no apparent fluorescence changes, demonstrating the high specificity of the strategy (Figs. 3A and 3B). These results exhibited that the Exo-RPA test had sufficient specificity to segregate the target TeNT gene from the genomes of common infectious bacteria, effectively minimizing the number of false-positive results.

RPA and PCR were performed to measure the extracted genomic DNA (ATCC 19406, NCTC 5405). Fig. S2 and Table S2 detail the outcomes of these two assays. After confirming the absence of inhibitors, ATCC 19406 genomic DNA was not amplified in either of the assays, while NCTC 5405 exhibited significant amplification by both methods. The above results indicated that both methods could distinguish between toxic and nontoxic strains of *C. tetani*. All the mass spectrometry results of bacteria are presented in Fig. S3.

### Repeatability and Different Volumes of the Assay

**Fig. 4:**
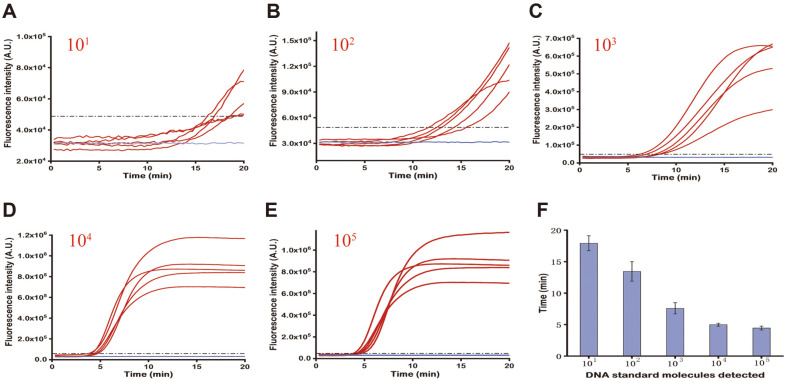
Repeatability of the RPA reaction. (**A-E**) RPA amplification of the TeNT gene. The concentration of the target in each sample is identified in the top left of each section. Red line: target amplification curves; blue line: no target control; black dashed line: fluorescence threshold. Only fluorescence intensity that exceeds the detection threshold is identified as positive. (**F**) Histogram indicating the average threshold time required for different concentrations of the target. The error bars signify the standard deviation of five measurements.

To validate the repeatability of the RPA approach, the RPA reactions were independently repeated five times for the target copy numbers ranging from 10-10^5^ copies/μl (Figs. 4A-4E). Strong fluorescence signal changes were discovered for the number of molecular targets from 10^2^ copies/μl in every independent experiment, and there was a slight difference between replicates, whilst the results for 10 copies/μl were more variable. A histogram with an error bar indicates the average threshold time required for different concentrations of the target (Fig. 4F). The RSD varied from 4.6% to 11.7%, which suggested acceptable reproducibility of the proposed RPA assay. The final fluorescence values for the same copy numbers denoted a relatively large difference, indicating that copy number could not be quantified precisely by the basic RPA assay. We tested the effect of different volumes (10 μl, 15 μl, 50 μl) on the experimental results. From Fig. S4, we could conclude that even when the reaction volume of RPA was reduced, the experiment could still be carried out smoothly. In addition, there was no large difference in reaction time.

### Detection Performance in Real Specimens

No complete suppression of the response was presented when serum was added to the RPA reaction tube, but an approximately tenfold reduction in sensitivity was obtained. This demonstrated that the RPA reaction could be inhibited by the biological factors that existed in the serum. Low-concentration targets were tested by spiking extracted pus samples to verify the development of biological sample analysis. The results revealed that there was no difference between the standard samples and the pus samples. Overall, our results preliminarily revealed the potential diagnostic value of the proposed RPA method for clinical sample analysis, which can be investigated further.

## Discussion

A real-time PCR assay for the diagnosis of tetanus was conducted by D. Akbulut [20], who reported a detection limit of 92 genomes in soft tissue infections among illicit drug users. In this experiment, the detection limit was 200 copies/reaction for conventional PCR. RPA is an innovative gene amplification technique that has been used in pathogen research in the development of disease symptoms. In this study, we portrayed a robust early Exo-RPA approach for the examination of *C. tetani*. The newly developed *C. tetani* method has a detection limit of 20 copies/reaction, which outweighs the conventional real-time PCR assay with higher sensitivity. RPA had a short turnaround time of less than 20 min, whereas both PCR and LAMP assay run times were approximately 60 min; the shorter run time of the RPA assay, ranging from 3 to 20min, was a major strength compared to the other two assays. Exo-RPA is a highly efficient amplification method and exhibits great potential to minimize the detection turnaround time. LAMP, introduced by Jiang [21], was analyzed through turbidimetry and electrophoresis on agarose gels with analytical sensitivities ranging from 10-10^6^ CFU/ml. According to E. Cohen [37], the gene encoding the neurotoxin was not identified in ATCC 19406. The results presented in our experiments also indicated that the TeNT gene is not present in ATCC 19406. This demonstrated that this standard bacterial strain could not be used to amplify the TeNT gene and that the results published by Jiang based on the ATCC 19406 might be incorrect. The indirect detection methods commonly used in LAMP, *i.e.*, turbidity and colorimetry, were not able to distinguish nonspecific amplification. The major LAMP-amplified products are a range of polymers, which appears as a “ladder” on the gel, and cannot be cloned and sequenced, hence restricting its practical widespread application for the molecular diagnosis of *C. tetani* [31]. However, RPA amplicons appear as a “single band” on gel and have been effectively proposed for the specific detection of various clinical specimens and different kinds of pathogens. High temperatures (> 90°C) are required for PCR and LAMP, whereas RPA can be completed at a constant 39°C and monitored by a smartphone with a signal detector [32]. LAMP requires four primers, and it is difficult to design primer pairs that work well [38]. Like real-time PCR, RPA requires only a single primer pair, and the probe is particularly selective for the targeting sequence, as expected. Higgins et al. designed a Python-based package (PrimedRPA) that can be used to efficiently design and screen a significant number of RPA primer and probe sets. This package can effectively reduce the time needed for experimental design [39]. Our assay was faster, had a relatively simple design, and did not require high-temperature incubation for a long time in advance of the amplification.

The primers were designed based on the conserved sequences showing specificity for *C. tetani*. An exo probe was introduced, and only when the probe matched the sequences properly was the THF cleaved to generate a fluorescent signal. This approach could decrease the nonspecific fluorescent signal compared to that generated in the basic RPA assay. There are two modified groups on the exo probe. The fluorophore and quenching groups were limited to modified thymine phosphoramidites, and the best performance is achieved when the separation distance between two groups is only 1-2 nt. Ole Behrmann and coworkers introduced an internal quenching group for the exo probe structure that eliminates the primary experimental design impediment. Due to the careful primer design, no evidence of cross-reactivity was observed with other bacterial genomes in our study. The results also showed that Exo-RPA had good performance in distinguishing between toxic and nontoxic *C. tetani* strains. There was no cross-reactivity with the nontoxic *C. tetani* strain in the RPA assay. The mass spectra and colony morphology observations are almost indistinguishable. The Exo-RPA assay successfully detected the target mixture of *C. tetani* with serum and extracted pus DNA, suggesting that RPA was a relatively stable and reasonably efficacious target amplification method applicable to clinical specimens. It was reported that an RPA commercial kit maintained at 45°C for up to 3 weeks was still capable of detecting targets, which showed that the RPA agents have long-term stability without requiring a cold chain and therefore could be applied in the primary rural government health departments or in survey fieldwork.

The RPA-based bacterial reporter system proposed in our research is a powerful new vehicle for rapid, precise, and sensitive quantification of bacteria in clinical samples, which might accelerate the diagnosis of tetanus.

## Supplemental Materials

Supplementary data for this paper are available on-line only at http://jmb.or.kr.

## Figures and Tables

**Table 1:** Primers and exo probe sequences used in the real-time RPA assay.

Name	Oligonucleotide sequence of designed primers (5′ to 3′)
TeNT gene RPA F1	TTGGAAGAAACTCTGAAGTAAAGGCCGGAAT
TeNT gene RPA F2	AAGAAACTCTGAAGTAAAGGCCGGAATAT
TeNT gene RPA F3	ACAAAACAGACCAAAGCATCAAGCAAGAAGT
TeNT gene RPA R1	TCAGTTTTGATGATATTTTGCCATGCTTCC
TeNT gene RPA R2	TTGTGTTCCTTAAAAAATAATGCTGTGCT
TeNT gene RPA R3	TGATAACAACATAATTCAGGGAGAAAGTAC
TeNT gene RPA P	5'-CTTAGTTTATTGCCAGGAAACTTCTTGCdT (FAM)T(THF)
	AdT (BHQ1)-GCTTTGGTCTGTTTT-(C3 Spacer)-3'

F: forward primer; R: reverse primer; P: exo probe; dT (FAM): thymidine nucleotide carrying Fluorescein; THF: tetrahydrofuran spacer; dT (BHQ1): thymidine nucleotide carrying Black Hole Quencher 1; C3 Spacer: blocking elongation.

**Table 2:** The concentration of the targets, triple threshold time and mean values of threshold time were listed.

DNA copies/μl	Time (min)			Mean	SD

	1	2	3		
1	Neg	Neg	Neg	None	None
5	Neg	Neg	Neg	None	None
10	18.7	17.0	16.3	17.3	1.01
10^2^	13.0	12.7	14.3	13.3	0.69
10^3^	7.0	8.0	8.3	7.8	0.55
10^4^	5.0	5.0	4.7	4.9	0.14
10^5^	4.7	4.3	4.0	4.3	0.29
10^6^	3.3	3.2	2.8	3.1	0.22
10^7^	2.5	2.3	2.4	2.4	0.08
10^8^	1.9	1.7	1.7	1.8	0.09

Neg: negative; SD: standard deviation.

**Table 3:** Influence of the inhibitory factors (in clinical samples) on the performance of the *C. tetani* RPA assays.

Concentrations (copies/μl)	RPA threshold time (min)		

	Standard sample	Serum	Pus extracted
10	17.3	Negative	18.0
10^2^	13.3	15	13.4
10^3^	7.8	8.33	7.67
