# Supplementary material for: Rapid Detection of Clostridium tetani by Recombinase Polymerase Amplification Using an Exo Probe
Source: J Microbiol Biotechnol. 2021 Nov 24;32(1):91–8. doi: 10.4014/jmb.2109.09022 (PMC9628835; doi:10.4014/jmb.2109.09022)
Supplement: Supplementary file 1 [file jmb-32-1-91-supple.pdf]

J. Microbiol. Biotechnol.
https://doi.org/10.4014/jmb.2109.09022

Supplemental Materials

Table S1. TeNT gene sequences and PCR primers.

Name	Oligonucleotide sequence (5′ to 3′)
PCR F1	ATGCGCCATCGTATACTAAC
PCR R1	CCATCTTTCGGATAACCTACA
PCR P1	(FAM)AATAGATTCTTTTGTTAGATCAGGTGATT(BHQ1)
PCR F2	TATGTATTTGACAAATGCG
PCR R2	CTTTCGGATAACCTACAAT
PCR P2	(FAM)CATCGTATACTAACGGAAAATTGAATAT(BHQ1)
TeNT gene	ATAACAGATT ATATGTATTT GACAAATGCG CCATCGTATA CTAACGGAAA ATTGAATATA TATTATAGAA GGTTATATAG TGGACTAAAA TTTATTATAA AAAGATATAC ACCTAATAAT GAAATAGATT CTTTTGTTAG ATCAGGTGAT TTTATTAAAT TATATGTATC ATATAACAAT AATGAGCACA TTGTAGGTTA TCCGAAAGAT GGAAATGCCT TTAATAATCT TGATAGAATT

F: forward primer; R: reverse primer; P: probe. FAM: Fluorescein; BHQ1: Black Hole Quencher 1.

Table S2. The concentration of the targets, triple Ct numbers and mean values of Ct numbers were listed.

DNA copies/μL	Ct number			Mean	SD
	1	2	3		
10	neg	neg	neg	-	-
10^2	36.36	36.89	36.88	36.71	0.25
10^3	33.01	33.26	33.93	33.06	0.14
10^4	29.63	29.62	29.62	29.62	0.01
10^5	25.59	25.23	25.49	25.44	0.15
10^6	21.05	21.00	21.65	21.23	0.30
10^7	17.35	17.20	17.44	17.33	0.10
10^8	14.24	14.58	14.71	14.50	0.20

Neg: negative; SD: standard deviation.

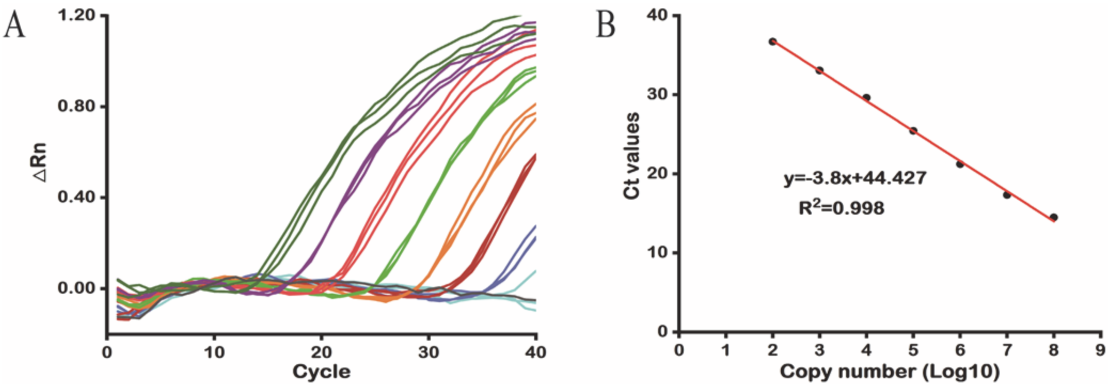

Fig. S1. Experimental sensitivity of the PCR. (A) The △Rn correlates with different concentrations of targets. From right to left: 10-10^8 copies/μL. Black line indicates the negative control. (B) The correlation between Ct and target gene content. From right to left: 10^2-10^8 copies/μL.

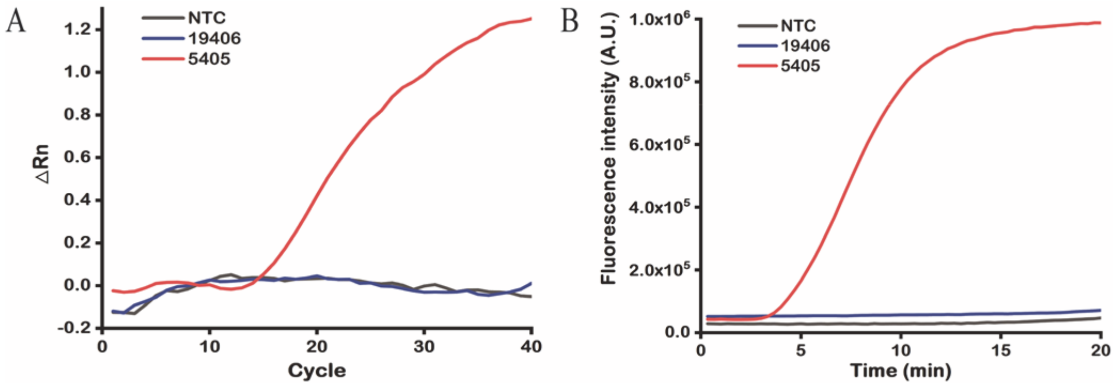

Fig. S2. Extracted genomic DNA detected. (A) Extracted genomic DNA was tested by PCR. (B) Extracted genomic DNA was tested by Exo-RPA. Black line indicates negative control, blue line indicates ATCC 19406, and red line indicates NCTC 5405.

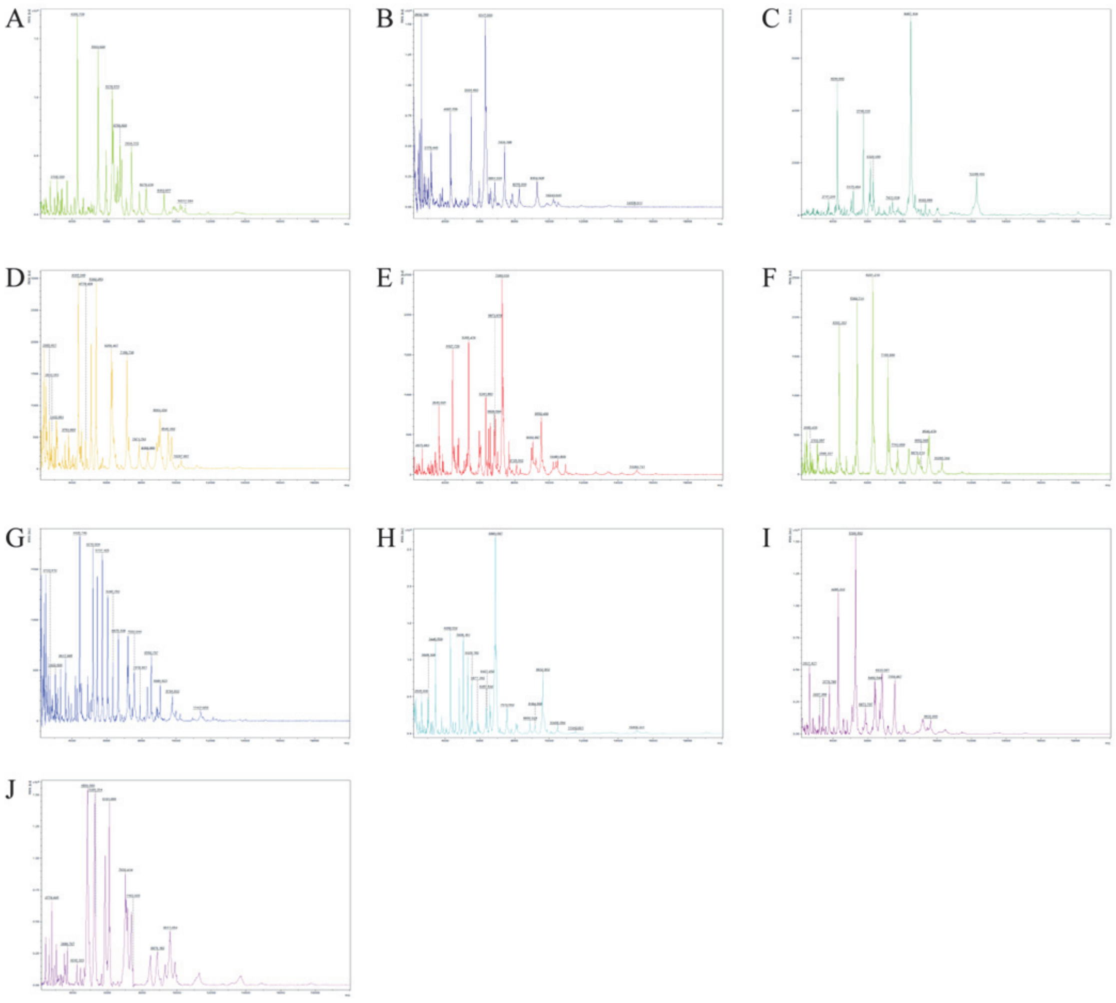

Fig. S3. Mass spectrometry results of bacteria. A-J: ATCC 19406, NCTC 5405, *A. baumannii*, *E. coli*, *E. faecium*, *K. pneumoniae*, *P. aeruginosa*, *S. aureus*, *S. epidermidis*, and *S. maltophilia.*

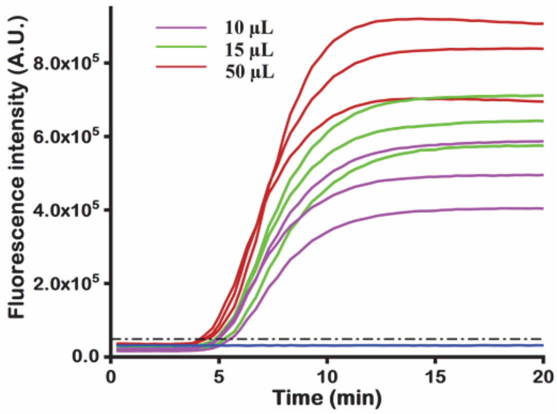

Fig. S4. RPA assays performed in different volumes.
